# Many Faces of Sleep: Diverse Sleep Characteristics and Their Joint Associations With Stress on Health in Adulthood

**DOI:** 10.1093/geroni/igab046.429

**Published:** 2021-12-17

**Authors:** Hye Won Chai, Soomi Lee, Nancy Sin

## Abstract

Two separate bodies of literature point to the significant roles of sleep and stress and their associations with health outcomes in adulthood. To further extend the field’s knowledge on sleep, stress, and health, it is essential to consider the multi-dimensional aspects of sleep and diverse stress contexts and identify ways in which the three factors are interrelated to each other. Different sleep characteristics may have varying implications for stress processes that, in turn, shape health outcomes. Therefore, this symposium integrates diverse characteristics of sleep (duration, quality, and pileup) in conjunction with various stress processes and experiences (bereavement, stressor exposure and appraisals, rumination), and examines their associations with cognitive, emotional, and physiological health outcomes. The first paper by Vigoureux and colleagues investigates the interaction between daily sleep and stressor frequency and severity on daily rumination. The second paper by Slavish and colleagues examines the bidirectional associations between daily stress and sleep duration and efficiency. The third paper by Mu and colleagues explores the mediated associations of sleep quality and sufficiency with work impairment through perceived cognitive abilities and rumination. The fourth paper by Lee uses the stress concept of pileup and tests how pileup of insufficient sleep is associated with day-to-day trajectories of affective and physical well-being. The final paper by Chai and colleagues examines whether sleep quality moderates the association between family bereavement and heart rate variability. The discussant, Dr. Nancy Sin, will integrate key points and discuss considerations for mechanisms and long-term implications of sleep, stress, and health.

